# Permeation of Phytochemicals of Selected Psychoactive Medicinal Plants across Excised Sheep Respiratory and Olfactory Epithelial Tissues

**DOI:** 10.3390/pharmaceutics15051423

**Published:** 2023-05-06

**Authors:** Anja Haasbroek-Pheiffer, Alvaro Viljoen, Jan Steenekamp, Weiyang Chen, Josias Hamman

**Affiliations:** 1 Centre of Excellence for Pharmaceutical Sciences (Pharmacen™), Faculty of Health Sciences, North-West University, Potchefstroom 2520, South Africa; anjahaasbroek11@gmail.com (A.H.-P.); jan.steenekamp@nwu.ac.za (J.S.); 2 Department of Pharmaceutical Sciences, Tshwane University of Technology, Private Bag X680, Pretoria 0001, South Africa; viljoenam@tut.ac.za (A.V.); chenw@tut.ac.za (W.C.); 3 SAMRC Herbal Drugs Research Unit, Tshwane University of Technology, Pretoria 0001, South Africa

**Keywords:** intranasal drug administration, ex vivo permeation, nose-to-brain drug delivery, excised sheep nasal tissue, *Centella asiatica*, asiaticoside, *Mesembryanthemum tortuosum*, mesembrine

## Abstract

The intranasal route of drug administration offers an opportunity to bypass the blood–brain barrier and deliver compounds directly into the brain. Scientific evidence exists for medicinal plants (e.g., *Centella asiatica* and *Mesembryanthemum tortuosum*) to treat central nervous system conditions such as anxiety and depression. The ex vivo permeation of selected phytochemicals (i.e., asiaticoside and mesembrine) has been measured across excised sheep nasal respiratory and olfactory tissue. Permeation studies were conducted on individual phytochemicals and *C. asiatica* and *M. tortuosum* crude extracts. Asiaticoside exhibited statistically significantly higher permeation across both tissues when applied alone as compared to the *C. asiatica* crude extract, while mesembrine permeation was similar when applied alone or as *M. tortuosum* crude extract. Permeation of all the phytocompounds was similar or slightly higher than that of the drug atenolol across the respiratory tissue. Permeation of all the phytocompounds was similar to or slightly lower than that of atenolol across the olfactory tissue. In general, the permeation was higher across the olfactory epithelial tissue than across the respiratory epithelial tissue and therefore showed potential for direct nose-to-brain delivery of the selected psychoactive phytochemicals.

## 1. Introduction

The intranasal (IN) route of drug administration has received increased attention as it offers an attractive alternative to oral drug administration [1]. IN drug delivery has advantages over other routes of drug administration, which include ease of administration, relatively high bioavailability, relatively fast onset of action and the possibility of bypassing the blood–brain barrier (BBB) [2]. Furthermore, systemic and/or direct nose-to-brain (N2B) drug delivery can occur after IN drug administration. The IN route is especially being investigated for the delivery of drugs used for the treatment of central nervous system (CNS) disorders in the brain. The most crucial regions for drug absorption from the nasal cavity are the olfactory and respiratory regions [3].

Several medicinal plant extracts and isolated phytochemicals have been studied for their therapeutic effects to treat CNS disorders [3,4]. Psycho-active effects that have been observed for medicinal plants include anti-convulsive, anti-depressant, anxiolytic, and neuroprotective activities amongst others [5,6,7]. Two plants often used in South Africa for their anxiolytic and anti-depressant actions are *Centella asiatica* and *Mesembryanthemum tortuosum* [7].

*Centella asiatica* (L.) Urb. (pennywort or gotu kola) belongs to the Apiaceae family and was originally found in tropical and sub-tropical parts of Asia and India but is now also naturalised in South Africa. Dried aerial parts of this plant have been powdered to be used as a snuff for its calming and sedative effects. Wanasuntronwong et al. [8] suggested that the γ-aminobutyric acid (GABA)-ergic mechanism is involved in its pharmacological action. It was also shown that glutamic acid decarboxylase (GAD) was stimulated by components of this plant, which is an enzyme involved in the synthesis of GABA. The most important group of biologically active compounds in this plant is the pentacyclic triterpenes, which include phytochemicals such as asiaticoside, asiatic acid, madecassoside, and madecassic acid [9]. Asiaticoside has shown promising antidepressant-like effects when used as a treatment on its own [10]. Several in vivo and clinical studies have shown the anxiolytic and anti-depressant activity associated with *C. asiatica* extracts [8,10,11,12,13,14,15,16]. To the knowledge of the authors, no pharmacokinetic or permeation studies across nasal epithelial mucosa have previously been reported for *C. asiatica*.

*Mesembryanthemum tortuosum* (L.) (also known as *Sceletium tortuosum* (L.) N.E. Br.; kanna or kougoed) is a succulent indigenous to South Africa belonging to the Mesembryanthemaceae family, which is widely used by traditional healers for its psycho-active properties against disorders such as depression, anxiety, bulimia, drug dependence and obsessive-compulsive disorder [17]. Dried *M. tortuosum* plant material is usually chewed or smoked, while powdered plant material has also been snuffed. There are four main alkaloids present in *M. tortuosum* extract, namely mesembrenol, mesembranol, mesembrenone and mesembrine. *M. tortuosum* extracts have shown very promising anxiolytic-like activity in zebrafish [4] as well as serotonin transporter (SERT) and phosphodiesterase 4 (PDE4) inhibitory activity [18]. The SERT and PDE4 activity relates mostly to mesembrine and mesembrenone [18]. Several in vitro, in vivo, and clinical studies have shown the anxiolytic and anti-depressant activity associated with *M. tortuosum* extracts [4,18,19,20,21,22,23,24,25,26,27,28], but the permeation of mesembrine alkaloids have only been investigated across excised pig buccal, sub-lingual and intestinal tissues [29].

The respiratory region in the nasal cavity covers the largest part and is supplied with abundant capillaries with high blood flow, which provides a relatively large surface where drugs can be absorbed into the systemic circulation [30]. Drug molecules taken up into the systemic circulation must cross the blood–brain barrier (BBB) to reach the brain. On the other hand, the olfactory epithelium provides a platform for the absorption of drug molecules directly into the brain, while avoiding the BBB. The olfactory epithelium contains basal cells, microvillar cells, and sustentacular cells (or supporting cells), which are interspersed with olfactory receptor neurons [30] as well as mucus-secreting Bowman’s glands [31]. The olfactory neurons extend into the olfactory bulb through the cribriform plate [32]. Several pathways exist for drug molecules to be absorbed directly into the brain after IN administration, namely the olfactory nerve pathway, trigeminal nerve pathway, and olfactory epithelium pathway [32,33].

In this study, the ex vivo permeation of selected phytochemicals after the application of the phytochemicals alone as well as after the application of crude extracts of two selected medicinal plants (i.e., *C. asiatica* and *M. tortuosum*) was evaluated across excised sheep nasal respiratory and olfactory epithelial tissues (which has been shown to be highly similar to human epithelial tissue because of the presence of ciliated and non-ciliated cells, Goblet and basal cells, and serous glands [34]). These ex vivo permeation studies were conducted to determine the potential for effective delivery of the selected phytochemicals into the brain after IN administration for the potential treatment of anxiety and depression.

## 2. Materials and Methods

### 2.1. Procurement of Plant Material

Dried *C. asiatica* and *M. tortuosum* plant materials were purchased from Parceval (Pty) Ltd. (Wellington, South Africa). Retention samples for both plants were deposited at the Department of Pharmaceutical Sciences (Tshwane University of Technology, Pretoria, South Africa). The asiaticoside reference standard was bought from Industrial Analytical (Pty) Ltd. (Kyalami, South Africa), while mesembrine alkaloid reference standards were isolated and purified using high-speed counter-current chromatography (HSCCC).

### 2.2. Preparation of Crude Extracts and Isolation of Phytochemicals

#### 2.2.1. *Centella asiatica*

The dried arial parts of *C. asiatica* (500 g) were infused in 95% ethanol at room temperature and shaken for 24 h using a mechanical shaker and then filtered through filter paper. The infusion and filtration processes were repeated three times with the same plant material to maximize the yield. The combined filtrates were concentrated under vacuum using a rotary evaporator and the residue was freeze-dried for 48 h to provide a yield of 34.94 g of crude extract.

#### 2.2.2. *Mesembryanthemum tortuosum*

An acid-base extraction method previously published was used to produce a crude extract from the dried aerial parts of *M. tortuosum* plant material (500 g) [35]. The extract was freeze-dried for 24 h to produce a yield of 5.56 g of extract.

The fractions were separated by silica gel column chromatography and the mesembrine alkaloids in the resulting fractions were separated by HSCCC [35]. Firstly, the alkaloid extract was dissolved in chloroform and fractioned by silica gel column chromatography in a glass column (7.0 cm i.d. 75 cm) packed with 200 g Kieselgel 60. The fractions were eluted from the column by using a chloroform-methanol gradient (100.0:0.0, 99.5:0.5, 99.0:1.0, 97.0:3.0 *v/v*). The fractions obtained from the column were qualitatively analysed by thin-layer chromatography with chloroform-methanol-10% ammonia (90.0:10.0:0.1 *v/v*) as developing liquid. Only fractions containing compounds that reacted with the Dragendorff reagent (indicating the presence of alkaloids) were selected for further alkaloid separation.

The mesembrine alkaloid in the selected fractions was isolated by HSCCC, using a suitable solvent combination (i.e., *n*-heptane-methanol-ethyl acetate-1% ammonia; 1:3:1:3 *v/v*). The HSCCC instrument consisted of a Spectrum model, multilayer coil-planet J-type centrifuge, equipped with two preparative coils connected in series (wrapped with polytetrafluoroethylene (PTFE) tubing, 1.6 mm i.d., 142 mL total volume). A portion of the selected fraction was dissolved in a mixture of 2 mL of each of the lower and upper phases and filtered before injection into the HSCCC (operated in normal mode at 30 °C). The coils were first filled with the stationary phase, then rotation (1500 rpm) was started, and the mobile phase (upper phase) was pumped at 3.0 mL/min (130 psi) until the mobile phase alone exits the coils. At this point, the sample was introduced via the sample loop. The instrument ran for approximately 100 min before reversing the flow direction and beginning elution with the lower phase. The samples were introduced into the column manually with an injection valve with a 5.0 mL loop from Rheodyne (Rohnert Park, CA, USA).

### 2.3. Analytical Methods

#### 2.3.1. Fluorescence Spectroscopy

The permeation samples were analysed for Lucifer yellow (LY) content by fluorescence spectroscopy using a multi-mode detection platform plate reader (Spectramax^®^ Paradigm^®^, Molecular Devices, San Jose, CA, USA) at excitation and emission wavelengths of 485 nm and 535 nm, respectively [36]. The fluorescence spectroscopy method was validated for accuracy, linearity, limit of detection (LOD), the limit of quantification (LOQ), and precision (intra-day and inter-day) according to the ICH guidelines [37].

#### 2.3.2. Ultra-Performance Liquid Chromatographic Linked to Mass Spectrometry (UPLC-MS) for Analysis of Phytocomponents (Alone and in the Crude Extract)

The asiaticoside content in the *C. asiatica* crude extract as well as in the permeation samples was determined by means of an ultra-performance liquid chromatograph (UPLC) system (Waters, Milford, MA, USA). Separation was achieved with a Cortecs C_18_ column (150 mm × 2.1 mm, i.d., 1.6 μm particle size, Waters), which was maintained at 40 °C. The mobile phase consisted of 0.1% formic acid in water (A) and acetonitrile (B) at a flow rate of 0.3 mL/min and the gradient elution followed was 95% A:5% B for 1 min, then 45% A:55% B in 13 min, then 95% A:5% B for 0.5 min, keeping for 0.5 min, and back to the initial ratio in 0.5 min, equilibrating the system for 1.5 min, with a total running time of 17 min. The standard solutions and permeation samples were injected in the mobile phase with an injection volume of 2 μL (full-loop injection). Masslynx chromatographic software was used to collect and process the data.

The same parameters as above (i.e., column, elution gradient, and flow rate) were used for the UPLC mass spectrometer (MS) analysis. The MS was operated in the negative ion electrospray mode. The desolvation gas used, was N_2_ at a flow rate of 500 L/h, while maintaining a desolvation temperature of 350 °C. The capillary and cone voltages were set to 2450 and 46 V, respectively, while the source temperature was 100 °C. The data were collected between 100 and 1200 *m/z* and the mean was calculated during acquisition by using the independent reference lock-mass ions via the LockSpray™ interface to ensure mass accuracy and reproducibility. The asiaticoside concentration was analysed as a marker molecule when evaluating the permeation of *C. asiatica* crude extract across the different nasal regions.

The phytochemical composition namely the mesembrine alkaloid content (mesembrenol, mesembranol, mesembrenone, mesembrine) of the *M. tortuosum* crude extract and permeation samples was determined by using a UPLC-MS method (Waters, Milford, MA, USA). Separation was performed on an Acquity BEH C_18_ column (150 mm × 2.1 mm, i.d., 1.7 μm particle size, Waters), which was maintained at 40 °C. The mobile phase consisted of (A) 0.1% ammonium hydroxide in water and (B) acetonitrile, which was fed into the column at a flow rate of 0.3 mL/min, with the gradient elution as follows: 80% A: 20% B, changing to 10% A: 90% B in 8 min, keeping for 0.5 min and back to the initial ratio in 0.25 min. A 2 μL injection volume was used to inject the standard solutions and samples in the mobile phase (full-loop injection). Masslynx 4.1 chromatographic software was used to collect and process the data. The MS was operated in a positive ion electrospray mode. The desolvation gas used, was N_2_, with the desolvation temperature set to 350 °C at a flow rate of 500 L/h and the source temperature was 100 °C. The voltages for the capillary and cones were set to 3000 and 38 V, respectively. Data were collected in the range between 100 and 1200 *m/z*, and the LockSpray™ interface was used during data acquisition to ensure mass accuracy and reproducibility.

The UPLC-MS method was validated according to ICH guidelines by determining accuracy, linearity, precision, LOD, and LOQ [37,38].

### 2.4. Tissue Preparation for Permeation Studies

Sheep snouts (Merino or Dorper) were procured from a local abattoir (Potchefstroom, South Africa) after the animals were slaughtered for meat production. Approval for this study was granted by the North-West University Animal Care Research Ethics Committee (NWU-AnimCareREC, approval nr. NWU-00537-20-A5). Briefly, the nasal cavity was separated from the scull with a reciprocating saw and the ethmoid conchae (olfactory tissue) as well as ventral nasal conchae (respiratory tissue) were removed from the nasal cavity. The epithelial tissues were gently stripped from the underlying cartilage by blunt dissection and cut into approximately 1.0 cm × 2.0 cm strips to be mounted in Sweetana-Grass permeation chambers (permeation area 1.78 cm^2^). Dissected tissues were used in permeation experiments within 1 h after procurement of the sheep snouts. The mounted tissue strips were pre-incubated with oxygenated Krebs-Ringer bicarbonate buffer (KRB; prepared according to the product information provided by Sigma-Aldrich (St. Louis, MO, USA) for equilibration at 37 °C for 30 min before permeation experiments commenced.

### 2.5. Assessment of Excised Nasal Tissue Integrity by Permeation of an Exclusion Marker (Lucifer Yellow)

Lucifer yellow (LY) was used as an exclusion marker to confirm that the excised tissue stayed intact inside the Sweetana-Grass diffusion chambers for the 2 h duration period of the permeation experiments [36]. LY was also dissolved in KRB containing 5% *v/v* ethanol to determine if the ethanol used to dissolve the plant extracts caused any tissue damage or contributed to increased membrane permeation. After the tissue was mounted and the chambers assembled, 7 mL pre-heated (37 °C) KRB were added to both sides of the membrane, placed in the heated block, and connected to carbogen (95% O_2_/5% CO_2_) to equilibrate for 30 min. The LY solutions (50 µg/mL) with and without 5% *v/v* ethanol were added to the apical chamber and samples (200 µL) were withdrawn from the basolateral chamber at various intervals until 120 min. The samples removed were replaced after every withdrawal with an equal volume of pre-heated KRB. The LY permeation samples were analysed by fluorescence spectroscopy.

### 2.6. Ex Vivo Permeation Studies with Plant Crude Extracts and Isolated Phytocompounds

The permeation of asiaticoside after application of *C. asiatica* crude extract and asiaticoside alone (solutions equivalent to 653.88 µg/mL asiaticoside dissolved in KRB containing 5% *v/v* ethanol) was measured in both the apical-to-basolateral (AP-BL or absorptive) and basolateral-to-apical (BL-AP or secretory) directions [39]. For permeation in the BL-AP direction, 7 mL of the test solution was added to the basolateral chamber instead of the apical chamber, and samples were taken from the apical chamber.

Permeation of mesembrine after application of *M. tortuosum* crude extract and mesembrine alone (solutions equivalent to 40 µg/mL mesembrine dissolved in KRB containing 5% *v/v* ethanol) was measured in the apical-to-basolateral (AP-BL; absorptive) direction only. It was previously shown that the permeation of mesembrine alkaloids was not affected by active efflux transporters [29].

All samples acquired after permeation experiments were analysed by the validated UPLC-MS method.

### 2.7. Histological Examination of Excised Sheep Nasal Epithelial Tissues

Freshly excised sheep nasal epithelial tissues from the respiratory and olfactory regions as well as excised sheep nasal epithelial tissues at the end of the permeation experiments (i.e., tissues exposed to the experimental solutions for 2 h during the permeation studies) were histologically examined. All tissue samples were fixed in 10% neutral buffered formalin for at least 24 h. The tissue samples were then dehydrated in a series of ethanol solutions (70%, 80%, 90%, and 100%), immersed in xylene, and embedded in paraffin wax at 56 °C [40]. The paraffin wax tissue blocks were sectioned with a microtome (5 µm thick). The slices were mounted onto microscope slides, dried overnight in an oven (37 °C), and stained the next day with haematoxylin, Eosin Y, and Alcian Blue (Sigma-Aldrich). The microscope slides were imaged with ZEISS 208 colour camera connected to a Nikon E800 compound microscope (Nikon, Tokyo, Japan) using a 10× objective [41].

### 2.8. Data Processing and Analysis

The percentage of each compound permeated across the excised sheep nasal tissues was calculated from the concentration measured in the samples withdrawn from the acceptor chamber at each time interval. The following equation was used to calculate the percentage permeation in each sample:

(1) $$\%\mathrm{Permeation}=\frac{\mathrm{Drug}\;\mathrm{concentration}\;\mathrm{at}\;\mathrm{specific}\;\mathrm{time}\;\mathrm{interval}}{\mathrm{Initial}\;\mathrm{drug}\;\mathrm{concentration}\;\mathrm{applied}}\;\times \;100$$

The apparent permeability coefficient (P_app_) values were calculated from the percentage permeation across the epithelial tissues. P_app_ is defined as the flow rate of the compound for which permeation is measured into the acceptor compartment normalised to the surface area through which the permeation occurs and to the initial concentration in the donor chamber, assuming the starting concentration on the opposite side is zero [42,43]. The following equation was used to calculate the P_app_:

(2) Papp=dcdt(1A×60×C0)

where (dc/dt) represents the permeability rate (concentration/min), A is the diffusion area and C_o_ is the starting concentration of the different compounds that will be used.

The efflux ratio was calculated to determine the effect of P-gp related transport on asiaticoside and was calculated from the P_app_ values in two directions. The following equation was used to calculate the efflux ratio [44]:

(3) $$\mathrm{ER}=\frac{P_{\mathrm{app}}\left(\mathrm{BL}\text{-}\mathrm{AP}\right)}{P_{\mathrm{app}}\left(\mathrm{AP}\text{-}\mathrm{BL}\right)}$$

where P_app_ (BL-AP) represents the apparent permeability in the secretory direction and P_app_ (AP-BL) represents the apparent permeability in the absorptive direction.

All experiments were performed with at least three independent replicates (n = 3). To determine if statistical differences existed between the permeation (P_app_ values) across the respiratory and olfactory tissues, the data were analysed using GraphPad Prism version 9 (www.graphpad.com; Accessed on 28 July 2022). All data sets were subjected to the D’Agostino Pearson omnibus K2 test to establish the normality and homogeneity of the data distribution. For data sets that were not normally distributed, non-parametric Mann-Whitney U testing was applied to determine the difference between the two groups analysed. Statistical differences were considered significant when *p* ≤ 0.05.

## 3. Results

### 3.1. Validation of Analytical Methods

The results of the validation of the fluorescence spectroscopy analytical method (to measure LY concentration) and the UPLC-MS analytical methods (to measure the four mesembrine alkaloids and asiaticoside) are shown in Table 1.

All the validation parameters obtained for the fluorescence spectroscopy and UPLC-MS analytical methods adhered to the guidelines published by the International Council for Harmonization [37,38].

### 3.2. Plant Crude Extract Characterization

**Figure 1:**
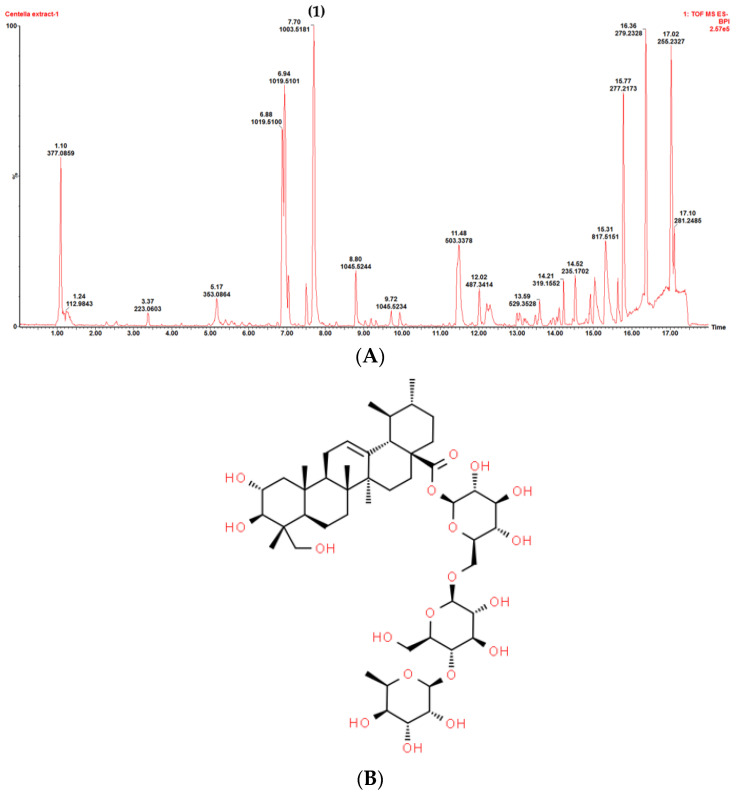
(**A**) Chromatographic profile obtained with UPLC-MS of the *Centella asiatica* ethanol extract. (1) peak for asiaticoside, and (**B**) the chemical structure of asiaticoside.

**Figure 2:**
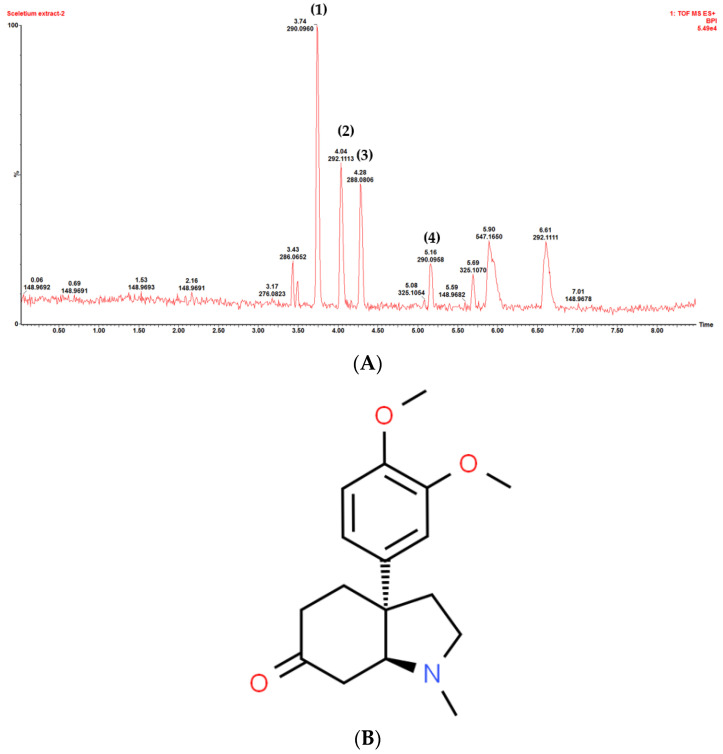
(**A**) Chromatographic profile obtained with UPLC-MS chromatogram of the *Mesembryanthemum tortuosum* acid-base extract. Peaks for (1) mesembrenol, (2) mesembranol, (3) mesembrenone, and (4) mesembrine, and (**B**) the chemical structure of mesembrine.

The phytochemical fingerprint profiles on UPLC-MS chromatograms of the *C. asiatica* ethanolic extract and the *M. tortuosum* acid-base extract are shown in Figure 1 and Figure 2, respectively. The asiaticoside content in the *C. asiatica* crude extract was found to be 149.5 µg/mg of dried material. The mesembrine content in the *M. tortuosum* crude extract was found to be 6.0 mg/g of dried material.

### 3.3. Assessment of Excised Nasal Tissue Integrity by Permeation of an Exclusion Marker (Lucifer Yellow)

The P_app_ values for LY across the respiratory and olfactory epithelial tissues were 0.53 ± 0.22 × 10^−6^ cm/s and 0.83 ± 0.35 × 10^−6^ cm/s, respectively. The P_app_ values for LY (dissolved in 5% *v/v* ethanol) across the respiratory and olfactory epithelial tissues were 0.26 ± 0.056 × 10^−6^ cm/s and 0.44 ± 0.032 × 10^−6^ cm/s, respectively. These P_app_ values for LY are below the value reported as an indication of an acceptable integrity of excised nasal epithelial tissues namely 1.72 ± 0.31 × 10^−6^ cm/s [45]. Furthermore, the 5% *v/v* ethanol did not result in an increased LY permeation, which indicated that the epithelial tissues were not damaged, and their permeability was not changed.

### 3.4. Ex Vivo Permeation Studies with Plant Crude Extracts and Isolated Phytocompounds

#### 3.4.1. Ex Vivo Permeation Studies with *Centella asiatica* Crude Extract and Isolated Phytocompound, Asiaticoside

**Figure 3:**
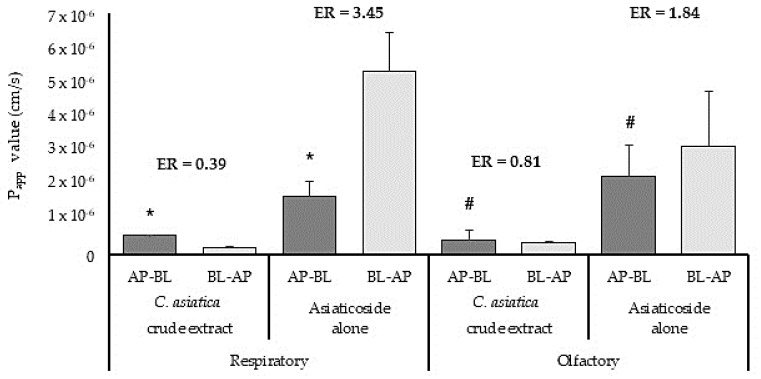
The apparent permeability coefficient (P_app_) values of asiaticoside across excised respiratory and olfactory sheep nasal epithelial tissues in the absorptive (AP-BL) and secretory (BL-AP) directions (n = 3, error bars = represent SD, ER = Efflux ratio). * P_app_ of asiaticoside is statistically significantly different between *C. asiatica* crude extract and asiaticoside alone across the respiratory tissue (*p* = 0.05). # P_app_ of asiaticoside is statistically significantly different between *C. asiatica* crude extract and asiaticoside alone across the olfactory tissue (*p* = 0.05).

The P_app_ values of asiaticoside after application of *C. asiatica* crude extract and asiaticoside alone across the excised sheep respiratory and olfactory tissues are shown in Figure 3. From this Figure, it is clear that the P_app_ value for asiaticoside alone in the absorptive direction across the respiratory epithelial tissue (P_app_ = 1.69 ± 0.45 10^−6^ cm/s) was statistically significantly higher than its permeation when the *C. asiatica* crude extract was applied (P_app_ = 0.55 ± 0.025 × 10^−6^ cm/s). Similar permeation results were obtained for asiaticoside across the olfactory tissue in the absorptive direction after application of the *C. asiatica* crude extract (P_app_ = 0.43 ± 0.28 × 10^−6^ cm/s) and asiaticoside alone (P_app_ = 2.28 ± 0.88 × 10^−6^ cm/s), which was also statistically significantly different. The permeation results of this ex vivo study are in agreement with the pharmacokinetic results obtained in an in vivo study reported by Hengjumrut et al. [46] where the bioavailability of asiaticoside was higher when administered as a single compound, than when it was administered as an extract to rats. Furthermore, the P_app_ values for both the asiaticoside alone and the *C. asiatica* crude extract across the respiratory tissue were similar to the P_app_ values previously reported for atenolol across respiratory (P_app_ = 0.67 ± 0.11 × 10^−6^ cm/s) and olfactory (P_app_ = 2.78 ± 0.36 × 10^−6^ cm/s) nasal epithelial tissues, a drug with poor to medium permeability [47].

In general, higher P_app_ values were obtained for the permeation of asiaticoside in the secretory direction than in the absorptive direction across the excised respiratory and olfactory tissue, which indicated it is subjected to active efflux transport. The efflux ratio for asiaticoside across the respiratory tissue (ER = 3.45) was higher than across the olfactory (ER = 1.84) tissue. This can possibly be explained by a lower expression of efflux transporters in the olfactory epithelium, but further investigations into transporter expression are needed to confirm this. The lack of asiaticoside efflux (ER < 1.0) when administered in the form of *C. asiatica* crude extract to the respiratory and olfactory tissues (Figure 3), suggested that there might be phytoconstituents in the crude extract which might have inhibited the active efflux transporters and thereby decreased the permeation of asiaticoside in the secretory direction [48].

#### 3.4.2. Ex Vivo Permeation Studies with *Mesembryanthemum tortuosum* Crude Extract and Isolated Phytocompound, Mesembrine

**Figure 4:**
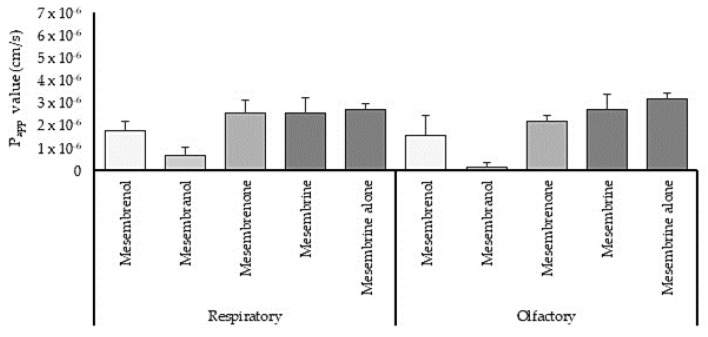
The apparent permeability coefficient (P_app_) values of the four mesembrine alkaloids after application of *M. tortuosum* crude extract and mesembrine alone across excised sheep nasal respiratory and olfactory epithelium (n = 3, error bars =represent SD).

Figure 4 shows the P_app_ values for the permeation of the four main alkaloids after application of *M. tortuosum* crude extract and for mesembrine when it was applied alone across the sheep respiratory and olfactory tissues. The permeation of mesembrenol, mesembranol and mesembrenone from the *M. tortuosum* crude extract was slightly higher across the respiratory epithelial tissue (P_app_ = 1.75 ± 0.38 × 10^−6^ cm/s, 0.63 ± 0.38 × 10^−6^ cm/s and 2.52 ±0.57 × 10^−6^ cm/s, respectively) as compared to their permeation across the olfactory epithelial tissue (P_app_ = 1.50 ± 0.90 × 10^−6^ cm/s, 0.11 ± 0.19 × 10^−6^ cm/s, 2.14 ± 0.29 × 10^−6^ cm/s, respectively). On the other hand, the permeation of mesembrine from the *M. tortuosum* crude extract when it was applied alone was slightly higher across the olfactory epithelial tissue than across the respiratory epithelial tissue. The permeation of the alkaloids in the *M. tortuosum* crude extract and mesembrine alone was similar or slightly higher than the permeation of atenolol across excised sheep respiratory tissue (P_app_ = 0.67± 0.11 × 10^−6^ cm/s), but slightly lower than the permeation of atenolol across excised sheep olfactory epithelial tissue (P_app_ = 2.78 ± 0.36 × 10^−6^ cm/s). Furthermore, the permeation of mesembrine after the application of the crude extract was similar to the permeation of mesembrine when it was applied alone. Therefore, the other phytochemicals in the *M. tortuosum* crude extract did not influence the permeation of mesembrine across the excised nasal epithelial tissues.

### 3.5. Histological Examination of Excised Sheep Nasal Epithelial Tissues

**Figure 5:**
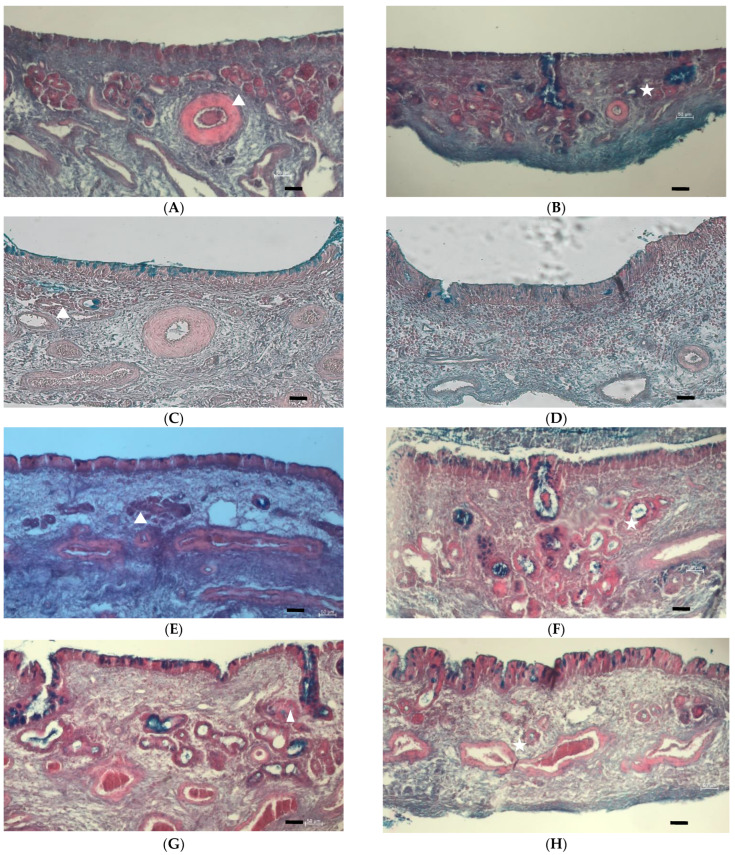

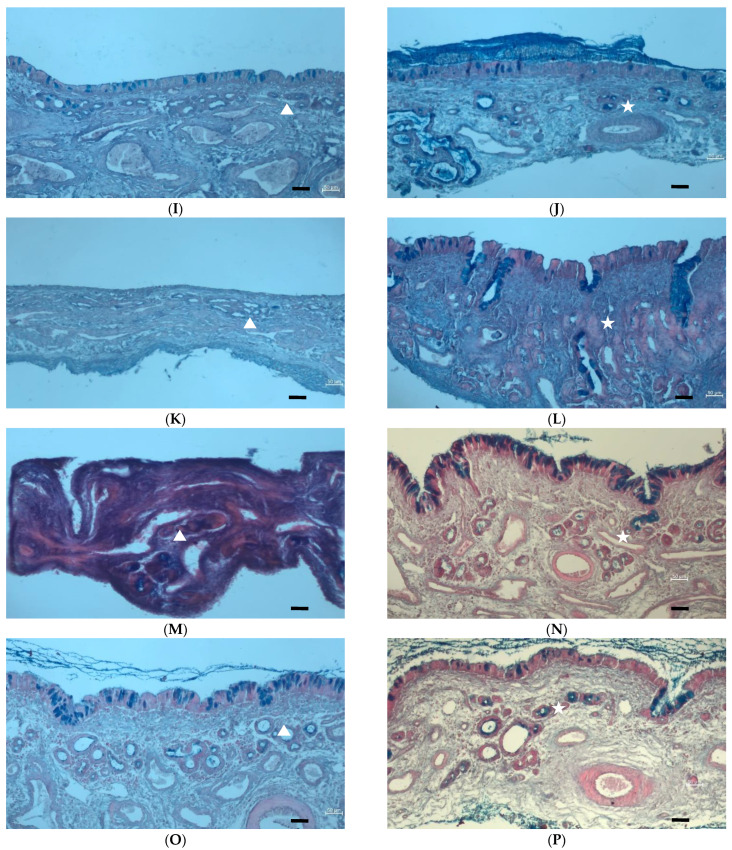
Histological micrographs of excised sheep nasal respiratory and olfactory epithelial tissues before and after treatment with selected plant extracts and phytochemicals. (**A**) Respiratory tissue before treatment; (**B**) Olfactory tissue before treatment; (**C**) Respiratory tissue after permeation of LY dissolved in 5% *v/v* ethanol; (**D**) Olfactory tissue after permeation of LY dissolved in 5% *v/v* ethanol; (**E**) Respiratory tissue after *C. asiatica* crude extract permeation in AP-BL direction; (**F**) Respiratory tissue after *C. asiatica* crude extract permeation in BL-AP direction; (**G**) Olfactory tissue after *C. asiatica* crude extract permeation in AP-BL direction; (**H**) Olfactory tissue after *C. asiatica* crude extract permeation in BL-AP direction (**I**) Respiratory tissue after asiaticoside applied alone permeation in AP-BL direction; (**J**) Respiratory tissue after asiaticoside applied alone permeation in BL-AP direction; (**K**) Olfactory tissue after asiaticoside applied alone permeation in AP-BL direction; (**L**) Olfactory tissue after asiaticoside applied alone permeation in BL-AP direction; (**M**) Respiratory tissue after *M. tortuosum* crude extract permeation; (**N**) Olfactory tissue after *M. tortuosum* crude extract permeation; (**O**) Respiratory tissue after mesembrine applied alone permeation; (**P**) Olfactory tissue after mesembrine applied alone permeation. The white triangles indicate the nasal glands and the white star indicates Bowman’s glands. Scale bars = 50 µm.

Figure 5 shows the micrographs obtained from the histological examination of the respiratory and olfactory tissues excised from the sheep nasal cavity before the permeation experiments were conducted (Figure 5A,B) as well as after a 2 h permeation experiment (Figure 5C–N). The tissue samples were stained with eosin (pink) to show the cytoplasm and extracellular matrix, haematoxylin (deep blue-purple) to show the nuclei, and Alcian Blue (bright blue) to show the mucus. In Figure 5, it can be noted that the presence of mucus (bright blue) is much more prominent on the olfactory epithelial tissue when the permeation experiment was conducted in the absorptive (AP-BL) direction (Figure 5D,H,L,N), whereas the mucus is located mainly in the Bowman’s glands within the olfactory tissue when the permeation was conducted in the secretory (BL-AP) direction (Figure 5 F,J). Mucus is secreted by respiratory and olfactory epithelium as a protective mechanism [49]. When stimulants are added to the basolateral side of the epithelium, the stimulants do not activate the cilia and goblet cells to secret mucus, the mucus stays localised in the goblet cells. When the excised nasal epithelial tissues at the end of the permeation studies (Figure 5 C–N) are compared to the epithelial tissues before permeation (Figure 5 A,B), the morphology is comparable without cellular changes that could be interpreted as tissue damage or toxic effects. From the histology micrographs, it is therefore clear that there was not a harmful effect on the tissue by the selected crude extracts or phytochemicals investigated in this study. This lack of tissue damage is comparable to previously published data regarding the safety of *C. asiatica* [50] and *M. tortuosum* [4]. The fact that the intact cilia can be seen on the apical side of the excised epithelium (Figure 5A–P) specifically shows a lack of damage to the ciliated columnar epithelial cells.

## 4. Conclusions

The permeation of asiaticoside after application of the *C. asiatica* crude extract was similar across the respiratory and olfactory tissues and there was no efflux noted. On the other hand, when asiaticoside was administered alone, it was actively effluxed in the secretory direction across both respiratory and olfactory epithelial tissues. This indicated that one or more of the coexisting phytochemical constituents in the *C. asiatica* crude extract inhibited the efflux of asiaticoside. To the knowledge of the authors, this is the first report on the permeation of the crude extract and the isolated phytocompound, asiaticoside, across nasal epithelial tissues. The permeation of mesembrine was only slightly higher across the olfactory tissue than the respiratory tissue. There was also no noticeable difference in the mesembrine permeation measured when administered as a crude extract than when it was administered as mesembrine alone. From the histological evaluations, no damage or toxicity was noted for any of the test solutions investigated. The results of this ex vivo permeation study indicated the potential for nose-to-brain delivery of phytochemicals from *C. asiatica* and *M. tortuosum* for the treatment of anxiety and depression. It is recommended that formulation strategies be investigated in future studies to improve the permeation of asiaticoside and mesembrine.

## Figures and Tables

**Table 1:** Regression analysis of calibration curves for asiaticoside, Lucifer yellow, and mesembrine alkaloids.

Analyte	R^2^	Regression Equation	LOD (µg/mL)	LOQ (µg/mL)	Accuracy	Precision (%RSD)	
						Intra-Day	Inter-Day
▪ Asiaticoside	0.9959	Y = 262X + 52	0.13	0.40	96.7%	1.81%	4.74%
▪ Lucifer yellow	0.998	Y = 229,4576,058X	0.012	0.036	99.2%	1.93%	1.42%
▪ Mesembrenol	0.993	Y = 2970X + 510	0.32	0.98	101.8%	0.96%	2.84%
▪ Mesembranol	0.989	Y = 4108X + 1067	0.23	0.71	98.7%	0.87%	1.59%
▪ Mesembrenone	0.995	Y = 2760X − 214	0.36	1.09	100.3%	0.44%	2.05%
▪ Mesembrine	0.993	Y = 4270X − 52	0.24	0.73	98.9%	1.40%	3.27%

## Data Availability

All materials, data, and protocols associated with the publication are available to the reader on request from the corresponding author.
